# Long-Term Care Services and Supports Needed for Successful Aging-in-Place: A Critical Review

**DOI:** 10.1146/annurev-publhealth-071823-113604

**Published:** 2025-04

**Authors:** Katherine E.M. Miller, Chase Mulholland Green, Abigail Fassinger, Jennifer L. Wolff

**Affiliations:** 1 Department of Health Policy and Management, and Roger and Flo Lipitz Center to Advance Policy in Aging and Disability, Bloomberg School of Public Health, Johns Hopkins University, Baltimore, Maryland, USA; 2 Hopkins Economics of Alzheimer’s Disease & Services Center, Johns Hopkins University, Baltimore, Maryland, USA; 3 Partnered Evidence-Based Policy Resource Center (PEPReC), Virginia Boston Healthcare System, Boston, Massachusetts, USA; 4 Division of Geriatric Medicine and Gerontology, School of Medicine, Johns Hopkins University, Baltimore, Maryland, USA

**Keywords:** caregivers, home care services, aging-in-place, independent living, health services

## Abstract

We summarize the state of the evidence on the long-term services and supports infrastructure to support aging-in-place. We find an extensive literature describing the importance of affordable medical and social services delivered in the home, support for family caregivers, and the role of technology for improving communication among patients, caregivers, and health care teams to support aging-in-place. We identify gaps in access to affordable services and an inadequate workforce prepared to support aging-in-place, raising concerns about meeting the future needs of our aging population. Interventions have been directed primarily at the individual level rather than at the system or policy level. Those systems-level interventions that do exist have been primarily within the health care sector: Little attention has been directed at developing holistic interventions that address multiple sectors, which reflects the lack of a cohesive public health delivery system for long-term services and supports nationally. Our findings collectively highlight the multifaceted nature of supports to enable aging-in-place and the necessity for future research to focus on drawing connections across domains of health services infrastructure.

## INTRODUCTION

The number of adults aged 65 and older in the United States is expected to exceed that of individuals under age 18 by 2034 due to population aging (129). Infrastructure domains—such as paid and unpaid personal care, health care, and social services—to support persons with physical and cognitive impairment who live in the community are known as long-term care services and supports (LTSS). Absent dramatic unexpected progress in therapies that alter the course of age-related physical and cognitive impairment, profound demographic shifts will yield unprecedented growth in demand for LTSS.

Most people desire to remain living at home at older ages (64). Because the United States has no national long-term care insurance system to cover services that help people remain at home, LTSS are often paid out of pocket or provided by unpaid family or friends (i.e., family caregivers). But LTSS are expensive, and privately financing LTSS is unattainable for most without incurring financial hardship (16, 42, 62, 77, 99, 143). Availability, accessibility, and preferences for LTSS and the ability to age-in-place vary by financial resources and also by family structure, race, ethnicity, gender, educational attainment, and geography (64). The interaction between individual socioeconomic characteristics and the physical and social environments over the life course contributes to notable inequities in LTSS access and quality. These inequities are compounded by underfunding, fragmented systems, and misaligned reimbursement incentives. In this context, aging-in-place while maximizing quality of life encompasses not only living in one’s home as independently and safely as possible but also minimizing financial hardship and imposing excessive demand on family or friends providing uncompensated care (i.e., family caregivers) (118).

The COVID-19 pandemic highlighted the importance of the physical and social community environments to aging-at-home with high quality of life (5, 7, 10, 12, 26, 32–34, 40, 47, 52, 54). Changes in cultural expectations, supported by regulatory and payment initiatives since the 1990s with the *Olmstead* decision, have shifted care from institutional settings, such as nursing homes, to the home and community (3, 57, 96). However, the prevailing literature has focused on specific aspects of the environment and services infrastructure, with less attention directed at comprehensively evaluating what is known about existing infrastructure to support LTSS in home-based settings and whether it is sufficient to meet the growing needs of an aging population.

This article first takes a broad view to conceptually describe domains for successful aging-in-place. We then review research on the effectiveness of programs and policies in key LTSS infrastructure domains. Finally, we discuss financing of health services infrastructure in the US context and highlight selected evidence-based care models to help older adults age-in-place.

## NEEDS OF OLDER ADULTS TO AGE-IN-PLACE

Aging-in-place refers to the ability to live in one’s setting of choice despite the onset or progression of impaired functional limitations. Thus, aging-in-place extends beyond remaining in one’s home to include individual values and preferences, available requisite supports, and an accommodating environment. Therefore, aging-in-place is a multidimensional concept that is the product of an individual person and their broader social and physical environments (73, 118).

The conceptualization and measurement of and expectations surrounding disability have profoundly evolved in ways that are relevant to aging-in-place. Prompted by advocacy and reinforced by legislation, conceptualization of disability has transitioned from a deficit orientation of assessing and supporting a person’s dysfunction toward recognizing that health and function exist on a continuum (3, 96). This approach, embodied by the World Health Organization (WHO)’s International Classification of Functioning, Disability, and Health (ICF), normalizes that impaired function is part of a broader human experience and that disability is the product of interactions between self and society. From the standpoint that disability reflects the absence of an accommodating environment (e.g., a sighted person without light cannot see), disability may be framed as a social issue that demands action (130). This evolution has inspired radical changes to social institutions across diverse sectors, including education, the workplace, and the built environment (e.g., ramps and curb cutouts), as well as a rebalancing of LTSS from institutions to the home.

**Figure 1:**
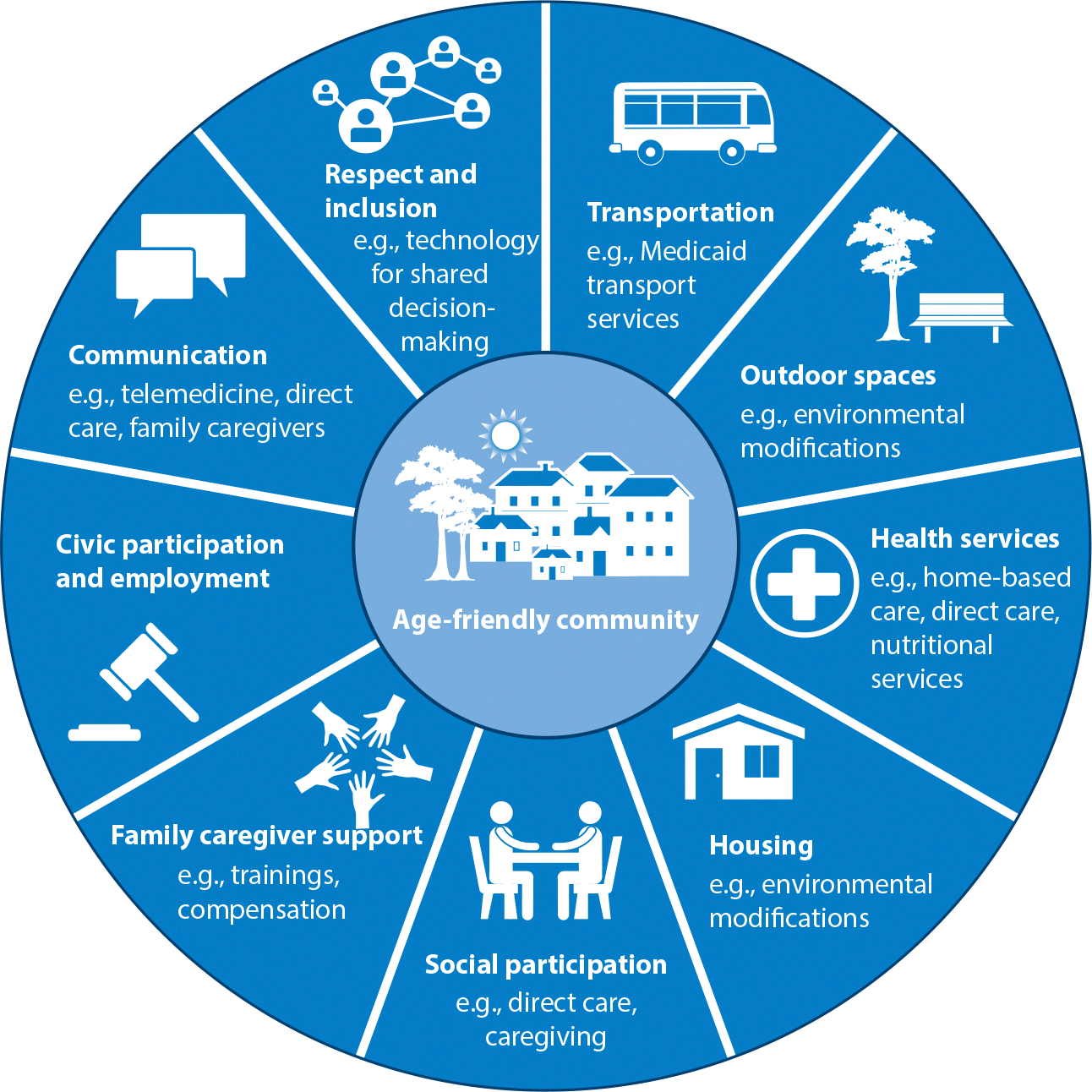
Adapted framework of infrastructure to support aging-in-place. Age-friendly community model from *Global Age-Friendly Cities: A Guide*, World Health Organization (140).

To organize this review, we draw on both the ICF and the WHO’s age-friendly communities initiative (140). This initiative embraces an aspirational goal in which any given person can age safely in a place that is right for them; be free from poverty; continue to develop personally; and contribute to their community while retaining autonomy, health, and dignity. The WHO initiative recognizes that multiple factors affect this goal and sets forth a framework with eight domains: (*a*) accessible and affordable health services, (*b*) adequate housing, (*c*) transportation, (*d*) outdoor spaces and buildings, (*e*) communication and information, (*f*) civic participation and employment, (*g*) social participation, and (*h*) respect and social inclusion. While each of the eight domains is independently important to aging-in-place, the framework explicitly recognizes the interplay between domains as well as across multiple levels of the environment, such as the family, household, neighborhood, community, and society. We further adapt the framework to include a ninth domain, support for family caregivers (Figure 1), which is particularly salient given our focus on infrastructure in the United States; 3 in 4 of older US adults with functional or cognitive impairment rely on family caregiver(s) for unpaid assistance (134, 139).

## EVIDENCE ON LONG-TERM SERVICES AND SUPPORTS FOR AGING-IN-PLACE

To identify evidence related to the existing LTSS infrastructure to support aging-in-place, we searched PubMed and Scopus for English-language studies of adults aged 65 or older from January 1, 2013, to June 30, 2023, using MeSH terms and terms from titles and abstracts. We identified search terms that captured home-based care, transportation, accommodating environments, environmental modifications, family caregiving, direct care, nutrition, and technology for community-dwelling adults, as aligned with the domains in Figure 1. The initial search yielded 3,972 unique articles. Due to the large number of primary data articles and a robust pool of systematic reviews on health services infrastructure (e.g., family caregiver interventions), we constrained our focus to systematic or scoping reviews.

We extracted data from 96 systematic or scoping reviews using Covidence (article information, objectives, number of articles reviewed, which of the nine adapted age-friendly community domains the article addressed, key findings, limitations, and areas for future research). We identified whether each review examined one or more of five outcomes relating to (*a*) quality of life, (*b*) nursing home entry, (*c*) mortality, (*d*) financial hardship, and/or (*e*) adverse consequences of caregiving. Within each of the adapted age-friendly community domains (Figure 1), we further identified whether the study reported the presence of positive evidence with respect to each of the five outcomes. Finally, we supplement our review with seminal articles otherwise unaddressed through included articles, prioritizing studies that focus on the US health services infrastructure to reflect its distinct context of health care delivery and financing and social policies.

To set the stage for our critical review, we first summarize the quantity, strength, and directionality of findings for domains of our adapted framework. We then describe the overlap of included articles that address each adapted age-friendly community domain using a correlation matrix of domains examined within each article. The correlations provide insight about the relationships among domains examined in the literature; e.g., if an article examined housing, is it more or less likely to also examine the role of family caregivers? Next, we summarize conclusions from included articles across each domain and each of the five outcomes. Finally, we provide a narrative overview of conclusions from included review articles.

We observed considerable variability in the representation of included review articles across the nine age-friendly community domains. Most articles (88%) included a focus on accessible and affordable health services, and more than half (52%) examined support for family caregivers. A substantial proportion examined housing (31%) or communication and information (30%). Fewer articles addressed social participation (14%), respect and social inclusion (8%), outdoor spaces and buildings (3%), and transportation (2%). No articles examined civic participation and employment. Forty-four percent of articles discussed adults with dementia, including Alzheimer’s disease and related dementias, and/or family caregivers of adults with dementia.

Table 1 presents the proportion of included articles within each age-friendly community domain that identified a benefit for each of the five specified outcome categories: nursing home entry, mortality, financial hardship, consequences of caregiving, and quality of life/unmet care needs. We find limited evidence of rigorous studies that support the role of age-friendly community domains for any of the outcome categories. We find the strongest evidence on the benefit of accessible and affordable health services, followed by support for family caregivers, with significant heterogeneity in the quality of studies and evaluated outcomes.

**Figure 2:**
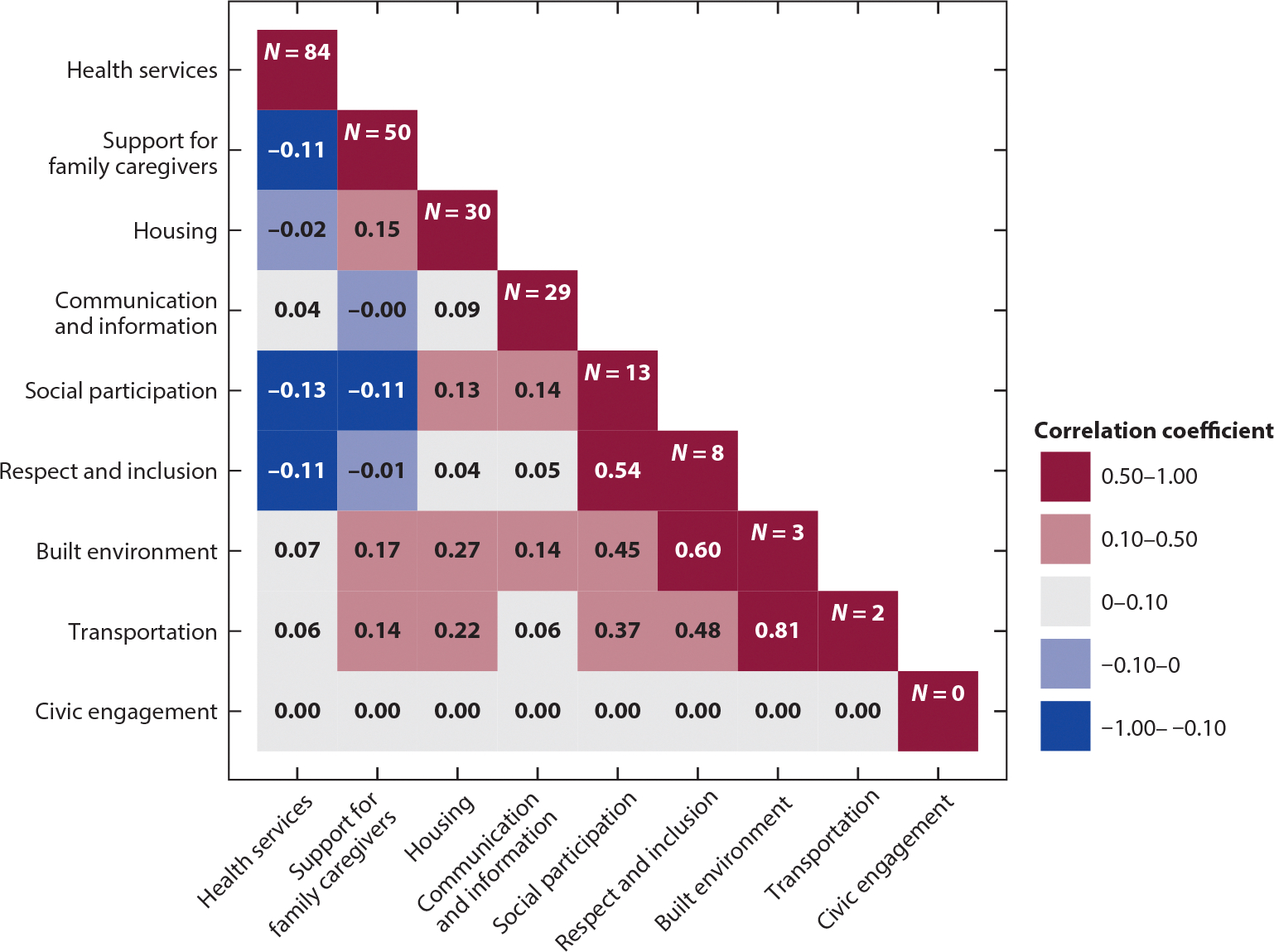
Correlation of review articles addressing adapted age-friendly community domains.

When evaluating the extent to which review articles addressed topics at the intersection of age-friendly community domains, we find weak correlation (absolute value of correlation coefficient <0.5) across accessible and affordable health services with family caregiver support, housing, and communication (Figure 2) (28). Review articles of social participation, respect and social inclusion, outdoor spaces and buildings, and transportation were more highly correlated (range of 0.54–0.81). Given these considerations, our critical review further collapses the nine domains into the following five groups: health services, support for family caregivers, housing/transportation/built environment, communication and information, and social participation/respect and inclusion/civic engagement.

### Health Services: Receiving Affordable Care in the Home

Health services span a wide spectrum of medical and social services, from home-delivered meals to primary and preventive services to acute care. Health services were the best-represented domain of our critical review (*n* = 84; 87.5% of identified articles). This domain also had the most evidence in support of improved aging-in-place outcomes. Included reviews found evidence that health services infrastructure benefited older adults’ quality of life or reduced unmet needs (*n* = 26/84 articles; 31.0%); reduced adverse consequences experienced by caregivers (17.9%); and reduced or delayed nursing home entry (15.5%), mortality (8.3%), and financial hardship (3.6%) (Table 1).

Medical and social services that assist individuals with personal care activities and remaining in the home are known as home- and community-based services (HCBS), which support aging-in-place. While aligned with patient preferences, evidence of existing HCBS programs that provide a constellation of services (e.g., personal care, home-delivered meals) for low-income adults is mixed; some evidence suggests increased hospitalization rates and identifies the need for greater integration of care (68, 144–146). In addition, access to and use of HCBS vary by geography, race, and ethnicity and can be exacerbated by the insufficient supply of workers to deliver care (11, 20, 21, 53, 63, 69, 88, 90).

Home-delivered meals are one example of an individual component of HCBS programs and can play a critical role in maintaining nutrition (18, 76) for the more than 10% of households with an older adult (aged 65+ years) who is food insecure (37). Meals on Wheels is one home-delivered meal program administered as a federal–state partnership through Area Agencies on Aging with modest annual funding from the Administration for Community Living by the Older Americans Act (38). Home-delivered meal programs reduce loneliness and nursing home placement and are correlated with positive patient outcomes, but they are underfunded and heterogeneous, necessitating additional research to better understand program efficacy and the potential value of broader scaling (17, 18, 55, 76, 101, 126–128).

A broad and growing range of medical care and social services can be provided in the home due to technology and communication innovations. Home-based primary care (HBPC) is one example of a home-based program that delivers care to older adults with complex chronic conditions and functional limitations who have difficulty traveling to a physical clinic. HBPC programs deliver and coordinate comprehensive medical care through intensive care coordination efforts in the home setting, commonly provided through a care team. An emerging body of evidence finds that HBPC reduces caregiver burden, hospital admissions, duration of hospital visits, and nursing home entry while improving patients’ quality of life (6, 59, 112, 123). HBPC continues to grow nationally with more than 40,000 providers and 4.4 million patients, yet access to the program is variable, with more limited supply in rural areas due to clinician shortages and transportation considerations (75, 109, 147, 150).

Skilled home health care (e.g., speech, occupational and physical therapy) has also grown significantly (94). While home-based services are generally aligned with patient preferences (49), the evidence demonstrating whether skilled home health care or skilled nursing facility care is more effective for reducing hospitalization readmissions is mixed (4, 79, 120, 138), undoubtedly due in part to the relevance of other home-based support, such as family caregiver availability and capacity. Similarly, home-based procedures such as dialysis are cost-effective, offer quality-of-life advantages (136), and are increasingly being used, yet they confer inequities in access and quality of care across race, socioeconomic status, Medicare Advantage plan, and rurality (41, 48, 78, 102, 136), which merit attention.

Technological innovation has also expanded the feasibility of delivering acute and specialty care in the home through telemedicine and remote patient monitoring. For example, hospital-at-home (HaH) programs can deliver hospital-level care in the home for specific conditions with lower risk for readmission and reduced depression and anxiety among patients (6, 22, 81, 98, 115). During the COVID-19 pandemic, the number of health care systems offering HaH programs grew rapidly, with 330 hospitals across 136 systems in 37 states in 2024 (23).

Despite the growing demand for health services to support aging-in-place, the evidence to date suggests that the workforce required is inadequate across the spectrum of health care providers—from geriatricians to nurses to direct care workers (36, 45, 63, 113). Direct care workers play a crucial role in HCBS delivery; yet while the workforce has grown, it has not kept pace with demand (63, 69). Recruitment and retention remain challenging, given the low wages, poor benefits, and potentially dangerous conditions (113, 114). In addition, few nurses are certified in geriatrics or have training to care for a geriatric population. In sharper contrast, the number of geriatricians has declined since 2010, and available fellowship slots remain unfilled, with adequate compensation as a primary challenge for recruitment (113).

Ultimately, the transition to providing care in the home has ramifications for individuals, care providers, and payers, as well as families, with potentially important unintended consequences stemming from an increased reliance on family caregivers (44, 67, 106–108).

### Supports for Family Caregivers

Establishing programs and policies to sustain and support family (broadly defined by each person) caregivers who provide health-related assistance to older adults living with chronic disease and disability is a critical consideration to achieving the optimal health care workforce for an aging population. Family caregivers are the predominant providers of LTSS and are thought to provide task assistance of the highest quality, at the lowest cost, and most consistent with older adults’ preferences. There is also a growing awareness among providers of the role that family caregivers play in ongoing, chronic care processes and potential benefits that may emanate from training and integration with the health care delivery team (Table 1) (29, 50, 86).

Although 50 included review articles addressed this domain, relatively few found rigorous evidence in support of improved aging-in-place outcomes. Included reviews found evidence that family caregiver support benefits older adults’ quality of life or reduced unmet needs (*n* = 13/50 articles; 26.0%); reduced adverse consequences experienced by caregivers (*n* = 13/50 articles; 26.0%); and reduced or delayed nursing home entry (*n* = 5/50 articles; 10.0%), mortality (*n* = 4/50 articles; 8.0%), and financial hardship (*n* = 1 article; 2%) (Table 1). While a prolific body of evidence examines the effects of family caregiving support, heterogeneity exists in the definitions of outcomes, interventions, and the ability to rigorously control for caregiver and care-recipient characteristics. This variation provides nuanced insights but complicates cross-study comparisons to inform recommendations for scaling.

Notable research effort has been devoted to developing training and support interventions to improve caregiver health and well-being, quality of life, and health care utilization (50, 51, 58, 91, 110, 122). The results from such interventions are heterogeneous and challenging to compare due to differences in the interventions being tested (e.g., multipronged versus single element), delivery mechanism (e.g., in-person, virtual, self-guided), and outcomes. Multicomponent, tailored, individual-level interventions demonstrate promise for improving caregiver outcomes, such as reduced burden or depressive symptoms (29, 58, 110). Strong and growing evidence also indicates that many or most caregivers have unmet training needs (14, 15, 142). However, scaling of interventions to address caregivers on a national scale is challenging, due, in part, to a lack of systematic identification of caregivers in care delivery settings and limited funding for caregiver support.

In addition to individualized interventions, policy is another mechanism to train, pay, and support family caregivers. In the United States, state and federal policies geared toward addressing caregiver needs are becoming more prevalent with the implementation of the National Strategy to Support Family Caregivers. For example, an increasing number of states are implementing paid leave programs. Four states offered paid leave prior to 2020; however, this number has expanded, and ten states have established or implemented paid leave programs as of 2024, although not all are active yet (8). Evidence of the effect of paid leave programs on family caregivers is limited but suggests some effect on increased caregiver labor force participation and mixed effects on caregiver health, well-being, and health costs (8). More than 40 states have implemented the Caregiver Advise, Record, Enable (CARE) Act to strengthen communication between health care providers and caregivers of hospitalized patients, which may improve patient experiences, but more evidence is necessary (71). In January 2024, the Centers for Medicare and Medicaid Services (CMS) implemented a billing code for health care providers to use to receive compensation for training family caregivers. These important advances in caregiving policy are tempered by low uptake in practice (84, 87).

The inadequate support available in the United States for family caregivers currently has important equity implications. As the intensity of caregiving, access to paid leave, and access to training from health care providers differ across dimensions of race, economic status, primary language spoken, and rurality, gaps in caregiver support may exacerbate existing inequities. The inaccessibility and limited supports for family caregivers are particularly pronounced for youth caregivers, i.e., individuals under age 18 who provide care to adults and/or siblings (72).

### Housing, Transportation, Outdoor Spaces, and Neighborhood

Housing, transportation, and the built environment are three interrelated domains of aging-in-place that are highly interwoven with medical and social services accessibility. Limited evidence surfaced for transportation and the built environment (*n* = 2 and 3 articles, respectively; Table 1). While 30 included review articles examined housing, identified effects for the 5 specified outcomes were limited, with the most support for improved quality of life or reduced unmet need (*n* = 4 articles; 13.3%).

Home and environmental modifications have a foundational role in closing the gap between individual capacity and environmental demands and are positively associated with psychological well-being (111). Modifications range widely, from simple and low-cost strategies, such as the installation of grab bars in showers or home monitoring technologies, to complex and costly companion robots (103, 117, 119). The acceptability and efficacy of different types of home and environmental modifications also vary widely. For example, older adults are more accepting of home monitoring systems that do not include video functions (103). The use of elder care technologies poses nonnegligible ethical and moral considerations to balance safety and care needs with autonomy and privacy (149). Sophisticated electronic assistive technologies, such as companion or rehabilitation robots, have been described as promising, with limited research in real-world settings, but are currently considered cost-prohibitive and acceptance is unknown (119).

Affordable and accessible housing that is equipped to meet the needs of persons with disabilities is lacking, and the dearth of units is expected to grow (85, 99). The rising costs of living and the growing share of older adults who rent (versus own) housing leave more older adults vulnerable to foreclosure or the inability to access affordable housing, respectively (43). Middle-income older adults are particularly at risk of financial hardship as most private insurances do not cover home-based care, and thus these households are less able to absorb the substantial costs of home-based care than are higher-income earners (99). Housing and health policy reform to address the unaffordability of housing for older adults specifically is necessary because housing and health are inextricably linked.

Few articles have examined the role of social factors in facilitating transportation and access to outdoor spaces and buildings (70, 80, 111). Yet rurality-based differences occur, with lack of transportation cited as a potential barrier to providing home-based care in rural areas (31, 80). Additional research is needed to understand how to leverage and scale transportation and built environment to support aging-in-place.

### Communication and Information to Empower Patients

Communication and information are the cornerstones of the formulation and execution of a care plan to support individual health. Technology innovation amplifies both opportunities for the increased volume and necessity of timely, transparent, accurate information exchange and communication among patients, families, and the care team. We identified 29 review articles in this domain. Evidence suggests that enhancing communication and information exchange through mechanisms such as e-health initiatives or Web-based trainings to support caregivers offers the potential to reduce or delay nursing home entry (*n* = 3; 10.3%), mortality (*n* = 3; 10.3%), unmet needs (*n* = 3; 10.3%), and adverse consequences of caregiving (*n* = 5; 17.2%).

Included review articles focus primarily on information exchange between some combination of the health care team, patients, and their family caregivers, with less attention to information sharing with community-based organizations. The role of digital health information and video conferencing was recognized as supporting aging-in-place as follows (35, 74, 148). E-health technology can facilitate shared decision-making through transparency, engagement, and information access across patients, family, health care teams, and social service providers and can deliver cognitive training, offer education, and build the skills and confidence of caregivers, including distance caregivers (74, 92, 95, 100, 137). Despite the prevalence of e-health technology use, engagement may erode over time, and major accessibility barriers exist (92, 137, 148). For example, people residing in rural areas without broadband Internet access and/or who have sensory or cognitive impairment may have reduced access and capability to use e-health technologies (1, 132). Proactive attention to usability and human-centered design, respect for individual autonomy and privacy, and the need to understand and respect the role of involved family caregivers were recurring findings across included articles.

While communication and information play key roles in facilitating aging-in-place, substantial variation exists in the quality and rigor of evaluations of specific interventions addressing outcomes directly related to aging-in-place. Since technology-based interventions often have multiple components, isolating the effectiveness and costs of individual components for cost-effectiveness analyses is challenging. Yet rigorous cost-effectiveness analyses of e-health technologies in real-world settings across different populations (e.g., by race, rurality, cognitive impairment) and the necessity for technologies to meet patients and families where they are (e.g., considering cognitive impairment) emerged as key areas of future research, with implications for implementation science striving to scale technologies to support aging-in-place.

### Social Participation, Respect, and Social Inclusion

In 2023, the US Surgeon General released a call for action to address the epidemic of loneliness and social isolation, as these experiences are linked to worse physical and mental health outcomes. As social isolation and loneliness may be triggered by retirement, widowhood, or functional decline, all of which are common with greater age, efforts to support social participation, respect, and social inclusion are imperative to aging-in-place initiatives. We identified 13 included review articles addressing social participation, with 8 addressing respect and social inclusion (Table 1). Few included articles identified effects on outcomes related to aging-in-place, and those that did identified a benefit for quality of life/unmet needs and reduced nursing home entry and mortality.

As care needs are dynamic, articles address the fundamental tension between preserving individual agency while meeting evolving care needs and fostering inclusion and participation (111). For example, the role of technology (e.g., digital storytelling) emerged as a potential intervention and mechanism to enhance independence and maintain freedom while facilitating family caregiver support to older adults, including long-distance caregivers (137). However, barriers exist for older adults to access technology and environmental modifications that support independence (as discussed above, e.g., privacy concerns) (124).

The empirical evidence of how social relationships affect health care use and, subsequently, aging-in-place is mixed (131). While social networks can impact patients’ use of home care, medical treatment adherence, and use of technology, the impacts of social networks on ambulatory care are ambiguous. The pathways by which different interventions impact social inclusion and respect are multifaceted due to variability in therapeutic elements and reliance on multipronged interventions. Further complicating the evaluation of the evidence is the inconsistent measurement of outcomes, as social isolation and loneliness are often conflated despite the capture of distinct outcomes and limited causal evidence of intervention efficacy in authentic home environments (95). While a consensus exists on the importance of social relationships and networks with regard to social isolation and loneliness, additional research from older adults and their caregivers is needed to develop a consensus on how to define and measure social inclusion as well as to further understand the pathways by which different types of interventions can impact patient outcomes (89, 111). The development of such a consensus would benefit evaluations for ongoing initiatives fostering social engagement in older adults. For example, the AARP Experience Corps trains and connects people over age 50 with third-grade students to improve students’ reading skills (27). When researchers pursue this agenda, ethical considerations of balancing patient desires and agency will be critical (125).

## FINANCING OF LONG-TERM SERVICES AND SUPPORTS AND INNOVATIVE CARE MODELS TO SUPPORT AGING-IN-PLACE

The infrastructure to support older adults seeking to age-in-place must be understood within the context of its financing landscape; here, we do so for the United States. Without a national long-term care insurance system, financing for LTSS, and HCBS specifically, is fragmented. The top three payers of LTSS are Medicaid (public insurance program for low-income individuals meeting income and wealth requirements; 44.3% of spending), Medicare (federal insurance program for adults primarily aged 65+; 19.8% of spending), and families paying out of pocket (13.6% of spending) (30). The substantial reliance on Medicaid has consequential implications for those who have access to insured HCBS.

### Medicaid

Medicaid finances HCBS, e.g., home care, telehealth, transportation, and support for caregivers (93, 105). Yet Medicaid reimbursement is notoriously low relative to private insurance and/or Medicare. Low reimbursement has important implications for access by eroding provider receptivity to delivering services to enrolled beneficiaries (2). Because Medicaid programs are jointly funded by the federal and state governments, significant heterogeneity exists across states regarding eligibility criteria and services offered. States fund HCBS primarily through 1915(c) waivers, which afford states flexibility to experiment with program characteristics, such as services offered, eligibility criteria, target population, and geographic areas served.

States have been at the forefront of developing innovative HCBS care models that integrate medical care, behavioral care, and HCBS to meet the needs of community-dwelling people with dementia (46, 90). For example, the Massachusetts 1915(c) Frail Elder Waiver provides coaching for families, in addition to a broader array of HCBS (e.g., home-delivered meals, homemaker and chore services) for people with dementia who meet eligibility criteria and are included in the program. Thus, access to Medicaid HCBS can vary widely both across and within states. Despite the intention of HCBS waivers to foster innovation, limited evidence of waiver effectiveness on person-centered outcomes exists (57). Moreover, an increasing number of states contract with managed care organizations to deliver Medicaid LTSS, which can obfuscate reported costs and use of Medicaid LTSS to CMS, thereby limiting evaluation of novel care models.

### Medicare

Medicare pays for nearly 20% of LTSS spending, primarily for postacute care (e.g., services provided short-term after a hip-replacement surgery), rather than services to support nonacute (maintenance) care needs. However, through the Center for Medicare and Medicaid Innovation, new payment models are being tested, evaluated, and, potentially, expanded to meet the needs of an aging population by bridging multiple domains of the adapted age-friendly community infrastructure necessary to age-in-place. For example, care models staffed by an interdisciplinary team of health care professionals (including direct care workers with clinical oversight) deliver comprehensive care in the home in combination with an alternate payment model from CMS.

Exemplar models include, but are not limited to, Program of All-Inclusive Care for the Elderly (PACE); Community Aging-in-Place—Advancing Better Living for Elders (CAPABLE); MIND at Home’s Guiding an Improved Dementia Experience (GUIDE) model; and Hospital-at-Home. For example, PACE is an integrated care model with capitated payments designed to help Medicare beneficiaries aged 55+ who require nursing home–level care to age-in-place. PACE enrollees are mostly dual-eligible for Medicare and Medicaid. PACE delivers comprehensive medical and social services to enrollees, primarily in adult day health centers through interdisciplinary care teams that include transportation, primary care, specialty care, home-based care, etc. PACE is associated with greater enrollee and caregiver satisfaction, fewer and shorter hospitalizations, fewer unmet needs, and lower mortality (9, 19, 56, 82, 83, 97, 116, 135, 141). However, scaling PACE and other exemplary care models requires identifying payment models to support implementation, which can be challenging given the fragmented financing and delivery of LTSS.

### Out of Pocket

Gaps in HCBS financing mean that individuals and families bear a notable burden of LTSS costs, and the individuals most likely to need home-based care are less likely to be able to afford it (62). This finding is notable, given that most adults aged 65+ do not have Medicaid coverage (92% in 2023). The private long-term care insurance market in the United States is small: Uptake has persistently been low (12–13% of adults aged 65+), few insurers offer LTSS coverage, and coverage is expensive (13, 24). Consequently, families may be incentivized to spend down assets in order to qualify for Medicaid and/or rely on unpaid care from family caregivers, which may confer negative consequences relating to physical, emotional, and financial well-being (25, 39, 60–62, 65, 66, 121, 133, 143).

The lack of systematic funding mechanisms to address services across age-friendly domains on a national scale exacerbates the disconnect between our long-term care system and the infrastructure required to support aging-in-place (e.g., affordable housing). For example, consider the funding for home-based meals; in fiscal year 2023, the total national budget for home-delivered meal programs funded by the Older Americans Act was $366 million, or 0.04% of total Medicare funding (104). The disconnect in levels of funding for acute/medical care and social services necessary for aging-in-place can impede the scaling of evidence-based care models and creation of the requisite workforce.

## CONCLUSION

Older adults with LTSS needs are heterogeneous and require a constellation of services and supports to address health and social and emotional well-being while continuing to live in their homes. In the United States, core aspects of the health services infrastructure are understudied, heterogeneous in defined interventions and outcomes, and lacking rigorous cost-effectiveness analyses. Evidence indicates an inadequate paid and unpaid workforce to meet demand and substantial barriers to accessing affordable health services in the home while preserving one’s autonomy, privacy, and preferences. Collectively, our review speaks to the multifaceted nature of supports to enable aging-in-place, which spans multiple domains, and the necessity for future research to focus on drawing connections across domains of health services infrastructure. A key challenge to scaling comprehensive supports relates to the varied funding sources and time horizons of requisite infrastructure both across and within domains.

The COVID-19 pandemic highlighted the fragmented, costly, and inadequate structure of LTSS and has moved Medicaid HCBS, provider reimbursement, and long-term care more broadly to the forefront of policy discussions. While federal policy has increased investments in Medicaid HCBS (e.g., the American Rescue Plan Act of 2021), the financing structure remains largely dependent on Medicaid and fails to address any meaningful financing structure for most older adults who will need to age-in-place and do not qualify for Medicaid. Gaps in the affordability and inaccessibility of key aspects of the infrastructure required to help individuals age-in-place (e.g., access to affordable high-quality HCBS, support for caregivers, transportation to/from health care providers) are cause for concern when considering how individuals in the United States will age-in-place as demand for care grows. While programs such as PACE and HBPC have been in existence since the 1970s and serve a relatively small number of individuals, integrated care models and services are receiving increasing attention as policy makers and legislators seek to address the expected needs of the aging population.

## Figures and Tables

**Table 1:** Percent of articles finding positive causal associations of health services infrastructure improving outcomes related to aging-in-place

*N* (row %)	Reduce/delay nursing home entry	Reduce/delay mortality	Reduce/delay financial hardship	Reduce/delay adverse consequences of caregiving	Quality of life/unmet care needs	Total number of articles (*N* = 96) *N* (% of total)
**Accessible and affordable health services**	13 (15.5%)	7 (8.3%)	3 (3.6%)	15 (17.9%)	26 (31.0%)	84 (87.5%)
**Support for family caregivers**	5 (10.0%)	4 (8.0%)	1 (2.0%)	13 (26.0%)	13 (26.0%)	50 (52.1%)
**Housing**	1 (3.0%)	1 (3.0%)	1 (3.0%)	2 (6.7%)	4 (13.3%)	30 (31.3%)
**Transportation**	1 (50.0%)	1 (50.0%)	0 (0.0%)	1 (50.0%)	1 (50.0%)	2 (2.1%)
**Outdoor spaces and buildings**	1 (33.3%)	1 (33.3%)	0 (0.0%)	1 (33.3%)	1 (33.3%)	3 (3.1%)
**Communication and information**	3 (10.3%)	3 (10.3%)	0 (0.0%)	5 (17.2%)	3 (10.3%)	29 (30.2%)
**Social participation**	2 (15.4%)	2 (15.4%)	0 (0.0%)	1 (7.7%)	3 (23.1%)	13 (13.5%)
**Respect and social inclusion**	1 (12.5%)	1 (12.5%)	0 (0.0%)	1 (12.5%)	1 (12.5%)	8 (8.3%)
**Civic participation and employment**	0 (0.0%)	0 (0.0%)	0 (0.0%)	0 (0.0%)	0 (0.0%)	0 (0.0%)

The denominator is 96, which is the total number of review articles. Percentages do not sum to 100 as articles may be counted under multiple domains of age-friendly communities as well as contribute to multiple objectives (e.g., reduce/delay nursing home entry).
